# Predictors of radiation-induced late rectal toxicity in prostate cancer treatment: a volumetric and dosimetric analysis

**DOI:** 10.3389/fonc.2024.1371384

**Published:** 2024-04-26

**Authors:** Simon K. B. Spohn, Gianluca Radicioni, Marcio Eisfelder, Constantinos Zamboglou, Dimos Baltas, Anca-Ligia Grosu, Ilias Sachpazidis

**Affiliations:** ^1^ Department of Radiation Oncology, Medical Centre – University of Freiburg, Faculty of Medicine, University of Freiburg, German Cancer Consortium (DKTK), Partner Site Freiburg, Freiburg, Germany; ^2^ Berta-Ottensein-Program, Faculty of Medicine, University of Freiburg, Freiburg, Germany; ^3^ Department of Radiation Oncology, German Oncology Centre, European University Cyprus, Limassol, Cyprus; ^4^ Division of Medical Physics, Department of Radiation Oncology, Medical Centre - University of Freiburg, Faculty of Medicine, University of Freiburg, German Cancer Consortium (DKTK), Partner Site Freiburg, Freiburg, Germany

**Keywords:** prostate cancer, radiation-induced toxicity, rectal toxicity, radiation-induced proctitis, dosimetric analysis, volumetric predictors

## Abstract

**Introduction:**

Prostate cancer (PCa) is a prevalent malignancy in European men, often treated with radiotherapy (RT) for localized disease. While modern RT achieves high success rates, concerns about late gastrointestinal (GI) toxicities persist. This retrospective study aims to identify predictors for late GI toxicities following definitive conventionally fractionated external beam RT (EBRT) for PCa, specifically exploring the dose to the rectal wall.

**Materials and methods:**

A cohort of 96 intermediate- to high-risk PCa patients underwent EBRT between 2008 and 2016. Rectum and rectum wall contours were delineated, and 3D dose matrices were extracted. Volumetric and dosimetric indices were computed, and statistical analyses were performed to identify predictors using the Mann–Whitney U-rank test, logistic regression, and recursive feature elimination.

**Results:**

In our cohort, 15 out of 96 patients experienced grade II late proctitis. Our analysis reveals distinct optimal predictors for rectum and rectum wall (RW) structures varying with *α/β* values (3.0 and 2.3 Gy) across prescribed doses of 68 to 76 Gy. Despite variability, RW predictors demonstrate greater consistency, notably V68Gy[%] to V74Gy[%] for *α/β* 3.0 Gy, and V68Gy[%] to V70Gy[%] for *α/β* 2.3 Gy. The model with *α/β* 2.3 Gy, featuring RW volume receiving 70 Gy (V70Gy[%]), stands out with a BIC value of 62.92, indicating its superior predictive effectiveness. Finally, focusing solely on the rectum structure, the V74Gy[%] emerges the best predictor for *α/β* 3.0 Gy, with a BIC value of 66.73.

**Conclusion:**

This investigation highlights the critical role of V70Gy[%] in the rectum wall as a robust predictor for grade II late gastrointestinal (GI) toxicity following external beam radiation therapy (EBRT) for prostate cancer (PCa). Furthermore, our findings suggest that focusing on the rectum wall specifically, rather than the entire rectum, may offer improved accuracy in assessing proctitis development. A *V70Gy (in EQD2 with α/β* 2.3 Gy*)* of ≤5% and if possible ≤1% for the rectal wall should be achieved to minimize the risk of late grade II proctitis.

## 1 Introduction

Prostate cancer (PCa) is the most frequently diagnosed malignancy among men in Europe. For patients who are diagnosed with localized disease, radiotherapy (RT) is a major definitive treatment option. Modern RT techniques and regimens reach high curation rates with biochemical recurrence-free survival rates (BRFS) of up to 92% after 5 years. Given the low prostate cancer-specific mortality, maintaining a good quality of life (QoL) and improving toxicities are of main interest for PCa patients and their treating radiation oncologists. Although late severe gastrointestinal (GI) toxicities are rare with rates of grade III toxicities of <2% according to the Common Terminology Criteria of Adverse Events (CTCAE), even grade II toxicities can severely impair the wellbeing of the patients.

Adding to existing body of evidence to reduce the risk of proctitis, this retrospective study aims to identify volumetric and/or dosimetric predictive factors for late GI toxicities after definitive conventionally fractionated external beam RT (EBRT). Additionally, we investigated whether the dose delivered to the sub-structure rectum wall is a more accurate predictor of toxicity than the dose delivered to the rectum.

## 2 Materials and methods

### 2.1 Patient cohort

In our retrospective study, we enrolled a total of *n* = 96 patients diagnosed with intermediate and high-risk prostate cancer according to the National Comprehensive Center Network (NCCN) criteria. The study was approved by the institutional review board of the University of Freiburg (Approval No.: 21-1149, dated 23 March 2021), and written informed consent was waived due to its retrospective nature. These patients underwent definitive conventionally fractionated EBRT using 3DCRT and/or IMRT techniques between March 2008 and September 2016, in our hospital. Definition of clinical target volumes (CTV) and planning target volumes (PTV) was performed according to current guideline recommendations. The prescribed dose in EQD2*_α/β=3.0Gy_* spanned from 68 to 82 Gy with a median of 74 Gy. Also, the number of fractions of the delivered plans varied from 34 to 46 with a median of 38 fractions. A summary of patient demographics and treatment details can be found in **Table 1**.

**Table 1:** Summary of characteristics in the prostate cancer cohort.

	Absolute value	Percentage (%)
**Number of cases**	96	100
Age (years)		
≤70	29	30
>70	67	70
Tumor classification		
cT1	09	09
cT2	59	63
cT3	27	28
PSA (ng/ml)		
<4	02	02
4–10	48	50
>10	46	48
Gleason score		
6	11	11
7	65	68
8–10	20	21
RT technique		
Volumetric modulated arc therapy (VMAT)	33	34
3D conformal radiotherapy (3DCRT)	26	27
Combi (VMAT, 3DCRT)	37	39
Treatment-related factors		
Hormonal therapy	52	54
Fiducial markers	90	94

The toxicity assessment was performed based on the electronic patient record and standardized follow-up documentation in our institution. Classification was done according to the CTCAE v5.0 criteria.

For our planning, we delineated both the rectum and rectum wall (RW) adhering to the guidelines established by RTOG (1). The RW has been extracted from the outer rectal contour subtracting 3 mm.

### 2.2 Method

The three-dimensional (3D) dose matrices for both single and summation plans were extracted from our Varian Eclipse treatment planning system (TPS) v15.6, utilizing the Eclipse Scripting Application Programming Interface (ESAPI). In the case of summation plans, the entire series of single plans was initially exported prior to any physical dose summation being applied. Subsequently, each dose matrix underwent conversion into the equivalent dose in 2-Gy fractions (EQD2), employing an *α/β* value of 3.0 Gy for both the rectum and rectum wall, in accordance with the linear quadratic model outlined in Equation 1. Additionally, we utilized an *α/β* value of 2.3 Gy for proctitis grade II or higher, as recently reported by Brand et al. (2).

We computed volumetric (relative and absolute) indices ranging from 20 to 80 Gy (in EQD2) at 1-Gy intervals (e.g., *V20Gy[%], V21Gy[%],…, V80Gy[%] and V20Gy[cc], V21Gy[cc],…, V80Gy[cc]*). Additionally, we calculated the dosimetric indices *D0.1cc*, *D2%* and *D1cc* to *D20cc* with an interval of 1 cc (e.g., *D1cc[Gy], D2cc[Gy], …, D20cc[Gy]*). The significance threshold was set at a p-value of 0.05. Subsequently, we subjected the two index families, volumetric and dosimetric index values, to analysis using the non-parametric Mann–Whitney U-rank test. Furthermore, we employed both univariate logistic regression analysis and a recursive feature elimination (RFE) process (3–5) to identify independent predictors and determine the most influential parameter associated with toxicity. In addition, for the analysis of the volumetric indices, we partitioned our cohort into five (5) risk groups, based on the prescribed dose in EQD2, specifically 68, 70, 72, 74, and 76 Gy. Model comparisons were conducted using Bayesian information criterion (BIC) accounting for the varying number of observations within each risk group; $BIC=k*\ln\left(n\right)-2\text{ln}\left(\hat{L}\right)$ , where $\hat{L}$ is the maximized value of the likelihood of the model, *n* is the number of the observations, and *k* is the number of parameters estimated by the model. Lower BIC values indicate better model and balance between model fit and complexity (6).

To explore potential correlations between covariates, such as patient age at the onset of treatment, PSA level, Gleason score, and androgen deprivation therapy (ADT), we conducted a multivariate logistic regression analysis. Our findings indicate that there is no significant correlation between grade II proctitis and the aforementioned covariates within our cohort. Specifically, there were no statistically significant associations observed with RT technique (p-value = 0.739), patient age (p-value = 0.748), PSA level (p-value = 0.500), Gleason score (p-value = 0.536), or hormonal therapy (p-value = 0.124). For the statistical analysis, the following python (v3.9.7) packages were utilized: sklearn v1.0.2, scipy v1.7.1, and statmodels v0.12.2.

### 2.3 Linear quadratic model

To convert the physical dose into the equivalent radiobiological dose in 2-Gy fractions (EQD2), we used the linear quadratic model (7, 8) as follows:


(1) $$EQD2=\;\frac{D\left(1+\;\frac{d}{\alpha /\beta }\right)}{1+\;\frac{2\;Gy}{\alpha /\beta }}$$

where *D* is the total delivered dose to the voxel, and *d* is the total dose delivered in *N* fractions (*d = D/N*).

## 3 Results

Ninety-six (96) patients with a follow up of 5 years were included in the final analysis. Fifteen (15) out of 96 patients experienced grade II late proctitis. Two (2) and one (1) cases with grades III and I late toxicity, respectively, were observed. The two cases with grade III late toxicity were excluded from our analysis.

**Figure 1:**
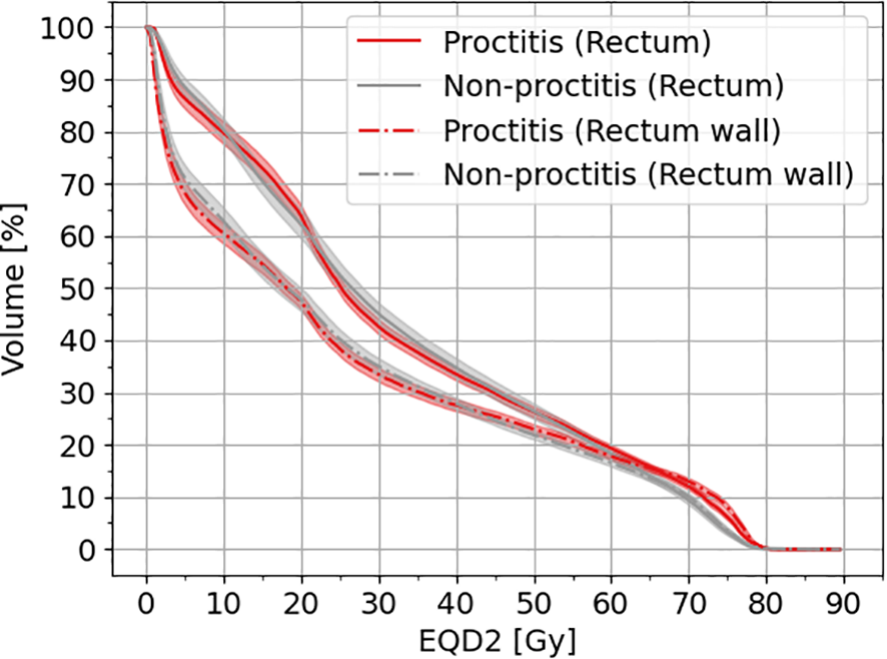
Cumulative DVH for rectum and rectal wall for patients with toxicity and non-toxicity. The shaded area indicates the 95% CI. EQD2 has been calculated with an *α/β* = 3 Gy.

In **Figure 1**, the cumulative dose–volume histograms (DVH) in equivalent dose in 2-Gy fractions (EQD2*_α/β=3.0Gy_*) are presented for both the rectum and rectal wall across two distinct patient groups: one experiencing toxicity (proctitis grade II) and the control groups. As illustrated in **Figure 1**, a noticeable line (red and gray) separation arises, particularly between 65 and 80 Gy in EQD2. This distinction implies a divergence in the cumulative dose distribution for the two groups suggesting a potential correlation with the onset of toxicity.

In our analysis of the rectum and rectum wall, we examined both volumetric (in relative and absolute values) and dosimetric parameters with a significance level set at p-value ≤ 0.05. In addition, we employed recursive feature elimination (RFE) for both the rectum and rectum wall (RW), and the results are summarized in **Table 2**.

**Table 2:** Predictors of late proctitis grade II toxicity for rectum and RW at two *α/β* values.

Structure	*α/β* [Gy]	Index family	Prescription dose [Gy]	Prediction parameter	BIC
Rectum	3.0	Dosimetric	Any (68, 70, 72, 74, 76)	D9%[Gy]	85.42
Rectum	3.0	Volumetric	68	V67Gy[%]	90.91
Rectum	3.0	Volumetric	70	V70Gy[cc]	89.05
Rectum	3.0	Volumetric	72	V70Gy[cc]	88.62
Rectum	3.0	Volumetric	74	V72Gy[cc]	88.62
Rectum	3.0	Volumetric	76	V74Gy[%]	66.73
RW	3.0	Dosimetric	Any (68, 70, 72, 74, 76)	D2cc[Gy]	83.53
RW	3.0	Volumetric	68	V68Gy[%]	88.14
RW	3.0	Volumetric	70	V68Gy[%]	88.14
RW	3.0	Volumetric	72	V68Gy[%]	88.01
RW	3.0	Volumetric	74	V74Gy[%]	73.56
RW	3.0	Volumetric	76	V70Gy[%]	63.09
Rectum	2.3	Dosimetric	Any (68, 70, 72, 74, 76)	D9%[Gy]	85.45
Rectum	2.3	Volumetric	68	V68Gy[%]	90.58
Rectum	2.3	Volumetric	70	V69Gy[%]	90.08
Rectum	2.3	Volumetric	72	V69Gy[cc]	88.68
Rectum	2.3	Volumetric	74	V69Gy[%]	79.29
Rectum	2.3	Volumetric	76	V72Gy[cc]	67.12
RW	2.3	Dosimetric	Any (68, 70, 72, 74, 76)	D2cc[Gy]	83.49
RW	2.3	Volumetric	68	V68Gy[%]	87.90
RW	2.3	Volumetric	70	V68Gy[%]	87.90
RW	2.3	Volumetric	72	V68Gy[%]	87.54
RW	2.3	Volumetric	74	V68Gy[%]	81.35
RW	2.3	Volumetric	76	V70Gy[%]	62.92

From our analysis, it is evident that various structures (rectum and rectum wall) and α/β values (3.0 and 2.3 Gy) lead to distinct optimal predictors across the prescribed doses ranging from 68 to 76 Gy. However, some consistency in predictors is observed within the same structure and *α/β* value across different prescription doses. Notably, the results for rectum wall (RW) appear to exhibit greater consistency across various prescription doses especially for *α/β* values of 3.0 and 2.3 Gy. For *α/β* of 3.0 Gy, the optimal volumetric predictors range from V68Gy[%] to V74Gy[%], while for *α/β* of 2.3 Gy, they range from V68Gy[%] to V70Gy[%]. Additionally, focusing solely on rectal structure results, V74Gy[%] emerges as the best predictor for *α/β* of 3.0 Gy with a BIC value of 66.73.

The best-fitted model, based on *α/β* of 2.3 Gy, exhibited a BIC value of 62.92. This finding indicates that the percentage of rectum wall (RW) volume receiving 70 Gy (V70Gy[%]) is the most effective predictor overall.

**Figure 2:**
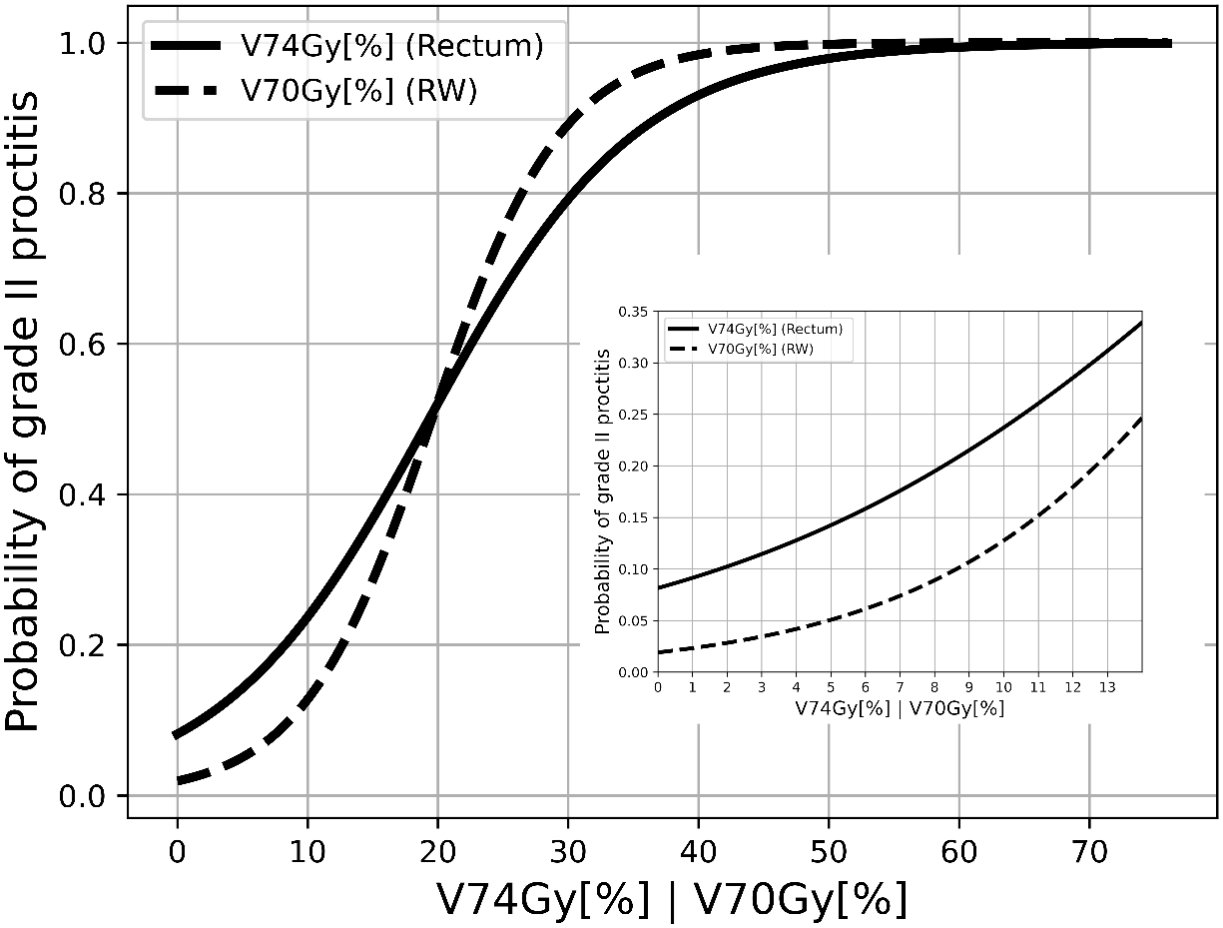
Response curves of late proctitis grade II against *V74Gy[%] in EQD2_α/β=3.0Gy_* for rectum and *V70Gy[%] in EQD2_α/β=2.3Gy_* for RW.

Employing univariate logistic regression analysis and representing the results as shown in **Figure 2**, we derived the following risk estimates for the rectum: a *V74Gy_(EQD2α/β=3.0Gy)_* below 1% was associated with a toxicity risk of 9.1%. Also, *V74Gy_(EQD2α/β=3.0Gy)_* values below 5% and 10% corresponded to a risk lower than 14.2% and 23.7%, respectively. For the rectum wall, *V70Gy*(*_EQD2α/β=2.3Gy)_* values below 1%, 5%, and 10% corresponded to a risk lower than 2.3%, 5.1%, and 12.7%, respectively.

## 4 Discussion

Modern dose-escalated EBRT for localized PCa yields high success rates with 5-year BRFS rates between 88% and 92% according to temporary phase III trials (9). Considering the high life expectancy, reduction of late toxicities become crucial to improve the QoL of patients. Despite various dose constraints existing for the rectum, optimal recommendations are still the subject of current research. This study identified additional volumetric predictors for late GI toxicity and demonstrated that considering the dose delivered to the rectal wall improves prediction.

We assessed GI toxicities according to CTCAE v5.0 (10, 11), as it represents the most contemporary reporting standard and is used in prospective clinical investigations for the characterization and grading of toxicity endpoints (12). However, former trials and analyses used solely characterization according to the Radiation Therapy Oncology Group criteria (RTOG), which hampers direct comparisons. In our study, we observed CTCAE grade II late rectal toxicities of 15.6%, which is in line with reports from recent phase III trials (13.7% according to RTOG in the CHHIP trial (13); 12.7% according to CTCAE in the FLAME trial (9)). A large retrospective analysis by Fonteyne et al. (14) with 637 patients and a median delivered dose of 74 Gy to the PTV demonstrated late grade II GI toxicity rates of 11.2% according to RTOG. Notably, this study included patients who received definitive and postoperative RT. Another study by Gullifold et al. (15) with 388 patients who received doses between 64 and 74 Gy with 3DCRT reported on higher rates with ≥ late grade II toxicities (RTOG) of 22.2%, whereas a study of Cuccia et al. demonstrated a late GI III toxicity of 11% for a cohort of 170 low-, intermediate- and high-risk prostate cancer patients, who received 70 Gy in 28 fractions to the prostate and 61.6 Gy to the seminal vesicles (16).

Landoni et al. (17) presented an overview of studies examining the correlation between dose–volume constraints for organs at risk and toxicity in prostate RT. To ensure that the incidences of grades ≥2 and ≥3 late rectal bleeding remain below 15% and 10%, respectively, the dose–volume constraints applied in treatment planning can be determined from various segments of the dose spectrum ranging from 40 to 75 Gy. Key constraints involve maintaining *V60Gy* < 35%–45%, *V70Gy* < 15%–25%, and *V75Gy* < 5%–10%. Peterson et al. (18) reported that the doses in the regions of 67.5 to 72.5 Gy are statistically significant predictors for late rectal toxicity (at 5 years, the cumulative incidence of late rectal toxicity was 37%, with only 5% being grade ≥2). In addition, Colaco et al. (19) performed a multivariate analysis of dosimetric and clinical factors to characterize gastrointestinal effects of proton therapy (rectal bleeding where grade ≥2, according to CTCAE v3.0, for a 2-year minimum follow-up) indicating that the volume of rectum or rectum wall receiving a dose equal or larger than 75 Gy is statistically significant with thresholds of 9.4% and 9.2%, respectively. Our results are also in line with QUANTEC constrains (risk of grade ≥2 rectal toxicity or rectal bleeding) *V74Gy* < 3% for 2 Gy per fraction, as well as with the constraints derived by Fonteyne et al. (14) for an equal or smaller than 10% probability of developing grade II or larger toxicity.

Groen et al. (20) analyzed the rectal doses within the recent phase III FLAME trial and confirmed a dose–effect relation for *D2cc* and *D50%* with a significantly higher risk of GI toxicities for higher doses. The authors used these selected dose parameters considering that *D2cc* is not influenced by the length of the anorectum and segmentation and D50% reflecting the middle organ dose. Notably, our modeling revealed that relative volumetric parameters exhibited the most significant association, while absolute volumetric parameters produced less relevant results. Brand et al. (21) demonstrated in a recent analysis of the CHHIP trial that the use of absolute-volume dosimetry or truncation of the rectum contour relative to the PTV did not result in statically significant differences. Our results are in line with these findings, since we found no improvement in toxicity prediction of absolute volumes compared to relative-volume analysis suggesting that relative-volume analysis is still preferable.

Furthermore, we found that delivered doses to the rectal wall improved toxicity prediction compared to considering the entire rectum. Overall, as depicted in **Table 2**, the rectal wall exhibits the smallest BIC for an *α/β* of 2.3 Gy.

A *V70Gy* of 1% of the rectal wall reduced the risk for grade II GI toxicities to 2.3%. Our results suggest that *V70Gy* for the rectal wall should not exceed 5% to limit the probability for QoL-impairing toxicities to 5%. Although there are only limited data on rectal wall dosimetry in the literature (18, 22, 23), considering the rectal wall appears plausible, since it represents the anatomical and functional boundary of the organ and thus provides a more accurate representation of the organ as risk exposed to radiation. Although Brand et al. (21) could not show a benefit for central rectal contour for toxicity prediction, accurate and standardized contouring might be more pronounced when analyzing substructures, such as the rectal wall.

Better understanding of the relationship between dosimetry of OARs and substructures with the clinical presentation of adverse effects is crucial to further improve RT in PCa. Particularly in modern therapy approaches, such as focal dose escalation based on advanced imaging modalities (9, 24, 25) and online adaptive planning workflows (26), deeper dosimetric analyses bear the potential to further improve dosimetry and reduce GI toxicities (26).

We acknowledge the limitations of our study. First, the sample size is relatively small. Second, approximately one-fourth of the patients were treated with 3DCRT only, which has to be considered, since IMRT is associated with more conformal treatment delivery and less dose to the rectum (27). Third, it is important to acknowledge that the data collection was retrospective, which inherently carries limitations regarding the quality of the data and may account for missing clinical information. Additionally, there is a possibility that other comorbidities could have influenced the occurrence of proctitis. However, it is noteworthy that our retrospective study lacked information on comorbidities such as diabetes, smoking habits, cardiovascular conditions, history of abdominal surgery, presence of inflammatory bowel disease (IBD) or diverticular disease, and anticoagulation usage. Yet, toxicities are standardized and documented using an electronic documentation system in our department. Last, patients were mostly treated with normofractionated RT regimens; consequently, extrapolation of our results to hypofractionated and ultra-hypofractionated regimens is limited. Nevertheless, we converted the physical dose into the equivalent radiobiological dose of 2 Gy.

## 5 Conclusions

We found a significant association between grade II late GI toxicity specifically proctitis with the *V74Gy*[%] in EQD2*_α/β_*_=3.0Gy_ for rectum and V70Gy[%] in EQD2*_α/β_*_=2.3Gy_ for rectum wall as the best predictor. Furthermore, our logistic regression model has demonstrated that the *V70Gy* of the rectal wall serves as a superior predictor by applying an *α/β* of 2.3 Gy. A *V70Gy* of ≤5% and, if possible, ≤1% for the rectal wall should be achieved to minimize the risk of late grade II proctitis. Our study adds additional constraints to already published recommendations. Future research should focus on anatomical substructures, such as the rectal wall, to improve calculation of normal tissue complication probabilities and further improve treatments.

## Data availability statement

The raw data supporting the conclusions of this article will be made available by the authors, upon reasonable request.

## Ethics statement

The studies involving humans were approved by Ethics Commission University of Freiburg Medical Centre. The studies were conducted in accordance with the local legislation and institutional requirements. The participants provided their written informed consent to participate in this study.

## Author contributions

SS: Conceptualization, Data curation, Methodology, Visualization, Writing – original draft, Writing – review & editing, Formal analysis, Investigation, Resources, Validation. GR: Data curation, Investigation, Writing – original draft, Writing – review & editing. ME: Data curation, Writing – original draft, Writing – review & editing, Investigation. CZ: Conceptualization, Data curation, Methodology, Validation, Writing – original draft, Writing – review & editing, Funding acquisition. DB: Conceptualization, Methodology, Writing – original draft, Writing – review & editing. A-LG: Methodology, Resources, Writing – original draft, Writing – review & editing, Funding acquisition. IS: Conceptualization, Data curation, Formal analysis, Investigation, Methodology, Project administration, Resources, Software, Supervision, Validation, Visualization, Writing – original draft, Writing – review & editing.
